# Supplementary material for: Chromothripsis during telomere crisis is independent of NHEJ, and consistent with a replicative origin
Source: Genome Res. 2019 May;29(5):737–49. doi: 10.1101/gr.240705.118 (PMC6499312; doi:10.1101/gr.240705.118)
Supplement: Supplemental Material [file supp_gr.240705.118_Supplemental_file_1.zip › contigs/annotated_contigs/DB114/contig.2.DB114_length_561_mean_cov_10.3172905526.docx]

**DB114_length_561_mean_cov_10.3172905526**

```
TGACAAGTTTCCCCAGCTCTCTCCCTCCTGCATGCATACAGGTGGGGTACTTTCTTACACATCTACATTGTCTTACGCATTTGCAGCGC
 >chr3:150620617-150620873 + E=2e-142
TCCACAGTCTGCCACTTAGAGAGTCTGTCCTATCCTACCTTCTATAGTGAGTTATTTGAATGTAACTCACTCCCTCAAATTTCACTGTC

AAATTGAAGAAGTGAATAAAGAGAGACTCAGAGTTTGGGAGGAAAGGGCTGTACTGCCACAAGTGTCCTTGAAG|AATA|TAACAAAGT
                                                                           >chr3:150240
CTTCTCGGAAAAATTCCTATAAAATCCAAGGTTAGAACATTTGGAAATAGCAAAAAAGATTATTGGGTATCTCAAATGGAAAACTAAGC
523-150240832 + E=2e-174
ACTATTTAGAACCCACCTTTCAGGTTATCTGATTGTACCTTTCCATTGCTTTTGAATTATTTTACAACTCTGTAAATTGTCAATAAGTG

ATAACAATGCAAAGTTAGTGTTGTTTACAAAGACATTTTGTTAGAGATCCAATTTTCTGCCCCCTGTTGAAAAAGTCAGTTTCTCCATC

CATTTGTAAATTCATTTCAGTATTGCTTT
```
